# Heavy metal pollution characteristics and health risk assessment of dust fall related to industrial activities in desert steppes

**DOI:** 10.7717/peerj.12430

**Published:** 2021-11-03

**Authors:** Zhe Xu, Wenbao Mi, Nan Mi, Xingang Fan, Ying Tian, Yao Zhou, Ya-nan Zhao

**Affiliations:** 1 College of Agriculture, Ningxia University, Yinchuan, Ningxia, China; 2 School of Geography and Planning, Ningxia University, Yinchuan, Ningxia, China; 3 West Development Research Center, Ningxia University, Yinchuan, Ningxia, China

**Keywords:** Desert steppe, Heavy metals in dust fall, Pollution characteristics, Health risks

## Abstract

China’s desert steppe is the transition zone between the grasslands in central China and the arid desert. Ecological security in this region has long been a subject of debate, both in the local and academic communities. Heavy metals and other pollutants are readily released during industrial production, combustion, and transportation, aggravating the vulnerability of the desert steppes. To understand the impact of industrial activiteis on the heavy metal content of dust fall in the desert steppe, a total of 37 dust fall samples were collected over 90 days. An inductively-coupled plasma mass spectrometer (NexION 350X) was used to measure the concentration of heavy metals Cu, Cd, Cr, Pb, Mn, Co, and Zn in the dust. Using comprehensive pollution index and multivariate statistical analysis methods, we explored the characteristics and sources of heavy metal pollution. We also quantitatively assessed the carcinogenic risks of heavy metals resulting from dust reduction with the help of health risk assessment models. The heavy metals’ comprehensive pollution index values in the study area’s dust fall were ranked as follows: Zn > Cd > Pb > Mn > Cu > Co > Cr. Among these, Zn, Cd, and Pb were significant pollution factors in the study area, and were affected by industrial production and transportation. The high pollution index was concentrated in the north of the research industrial park and on both sides of a highway. The seven heavy metals’ total non-carcinogenic risk index (HI) values were ranked as follows: Mn > Co > Pb > Zn > Cr > Cu > Cd (only the HI of Mn was greater than one). Excluding Mn, the non-carcinogenic and carcinogenic risk index values of the other six heavy metals were within acceptable ranges. Previous studies have also shown that industrial transportation and production have had a significant impact on the heavy metal content of dust fall in the desert steppe.

## Introduction

Due to accelerated social and economic development, an increasing number of pollutants are frequently discharged into the environment (Huang et al., 2015). Among these air pollutants, heavy metals are some of the most highly toxic, mobile, unstable, and accumulative (Koz, Cevik & Akbulut, 2012; Turkyilmaz, Sevik & Cetin, 2018). They are often loosely bound to soil and dust, eventually entering the soil-plant system over time, and endanger human health *via* accumulation in the food chain (Zhao et al., 2012a). Identifying the source of heavy metals is critical for preventing environmental pollution and protecting human health (Tang et al., 2013). Recent research on heavy metals found in dust fall has mainly focused on cities, parks, and roads (Taylor, 2015; Parvizimehr et al., 2020; Zheng et al., 2010). Direct dust sample collection methods using air samplers can be used to determine heavy metal concentrations per unit area in a short period of time (Mohsen, Ahmed & Zhou, 2018; Olawoyin et al., 2018), and indirect methods using dust tanks, collected rainwater, soil, plant leaves, or bark as biological indicators can be used to determine the long-term concentration of heavy metals in dust (Uchiyama et al., 2019; Miri et al., 2016; Gholizadeh et al., 2019). Such long-term analytic methods are simple, easy to operate, inexpensive, and low risk. Additionally, these approaches can effectively identify the concentration of heavy metals in collected samples (Gupta et al., 2016; Sawidis et al., 2011; Zhao et al., 2012a). Naderizadeh, Khademi & Ayoubi (2016) used biological indicator collection methods and found that Zn, Cu, and Pb in southwestern Iranian dust fall were mainly derived from human factors. Other studies have shown that heavy metal elements such as Cd and Pb are the main factors that cause air pollution (Avino, Capannesi & Rosada, 2008; Apeagyei, Bank & Spengler, 2011), heavy metals can damage human neurocognitive ability and increase the risk of cancer (Zhao et al., 2018), and long-term exposure to high concentrations of heavy metals can adversely affect human health and even cause death (Shaban, Abdou & Hassan, 2016; Zeng et al., 2016).

Desert steppe ecological security issues are a source of debate and the focus of an ongoing study on heavy metal pollution in desert steppe dust (Bayanmunkh et al., 2017; Zhao et al., 2012b). Because of the desert steppe’s unique geographical location, heavy metals in dust are easily re-distributed and enter the human body *via* hand-oral ingestion, skin contact, and inhalation, and can cause adverse health effects (Ewers, 1991; Atiemo et al., 2011; Wei et al., 2010a, 2010b; Wei et al., 2015). Additionally, they enter the soil through leaching and infiltration, accumulate in the food chain, and damage the health of the ecosystem (Hou et al., 2017). This phenomenon has become increasingly severe with rapid industrial development. Therefore, it is necessary to conduct research on heavy metals in atmospheric dust fall in the desert steppe, determine the source of these heavy metals, and reduce their input. This research is of great significance for preventing ecological degradation and maintaining the health of desert steppe ecosystems.

We selected China’s desert steppe as the object of this study and used dust suppression tanks to collect samples indirectly. The Cd, Cu, Pb, Zn, Cr, Co, and Mn contents in the dust suppression samples were then determined, and the pollution level, spatial distribution characteristics, and health effects of heavy metals were evaluated. The purpose of this research was to: (1) analyse the heavy metal pollution status of dust fall in the study area using the Nemeiro pollution, potential ecological risk, geo-accumulation pollution, and heavy metal comprehensive pollution indices; (2) determine the sources of heavy metals in dust fall *via* geostatistical, correlation, and positive matrix factorization model analyses; and (3) quantitatively assess the non-carcinogenic and carcinogenic health risks of heavy metals in dust fall. This study also aimed to explore the comprehensive characteristics of heavy metal elements in dust affected by industrial activities, identify the sources of pollution, and determine the health risk index values in order to provide references for the atmospheric environmental protection of desert grasslands.

## Materials and Methods

### Overview of the study area

The study area was located in west Gaoshawo Town, Yanchi County, Wuzhong City, Ningxia, China (106°49′6.18″E, 38°07′9.93″N). The terrain is high in the south and low in the north, and is dominated by hills with minor fluctuations in elevation. It is adjacent to Dingbian County in Shaanxi Province to the east, the Mu Us Desert (part of the Ordos Platform) to the north, and the Loess Plateau to the south. It is the transition zone between the grassland area of central China and the arid desert. The Industrial Park, comprised of the Thorning Dong Energy and Chemical Base, focuses on the development of coal, oil, gas, and new energy technologies. This region is situated along the Qingyin Expressway, 307 State Road, and the Taizhongyin railway and plays an important role in transport. Typically, this area has a continental monsoon climate with droughts, an annual average temperature of 7.8 °C, and an average temperature difference between summer and winter of approximately 28 °C. The soil types in this region are sandy and sierozem-based soils (Chinese soil taxonomy). The major vegetation types in the area include *Stipa breviflora* Griseb., *Agropyron cristatum* (L.) Gaertn., *Pennisetum centrasiaticum* Tzvel., *Lespedeza potaninii* Vass., *Potentilla chinensis* Ser., and *Artemisia scoparia* Waldst. et Kit.

### Sample collection and determination

**Figure 1:**
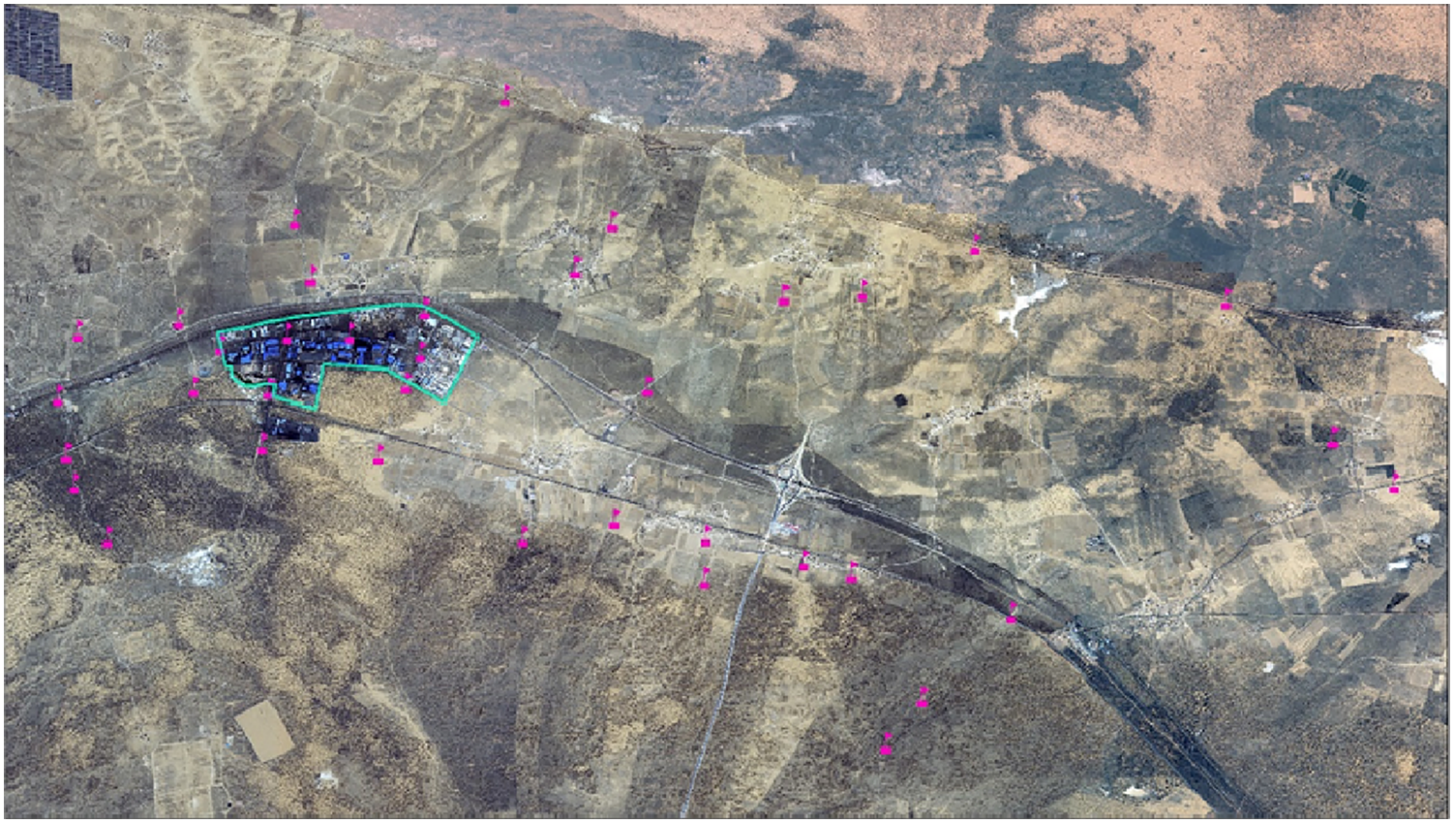
Map of the sampling points in the study area.

Using the grassland resource field survey method, we comprehensively considered the regional topography, climatic characteristics, and road traffic. We set up 50 sampling points in the study area with three dust reduction tanks at each sampling point to ensure that the collected materials could reach the test measurement amount. The sampling period was from 10 October 2019 to 10 January 2020. Continuous sampling was carried out for 90 days, and 37 effective sampling points were collected (Fig. 1). Atmospheric dust fall was sampled in accordance with standard GB/T 15265-1994 (‘Ambient Air Dust Fall Determination Gravimetric Method’) using a 15 × 30 cm plexiglass cylinder with a smooth inner wall and a flat bottom. There were no tall trees or buildings near the sampling point. Each point was fixed at a height of approximately 3–5 m to avoid pollution discharge areas such as chimneys. Before sampling, the plexiglass cylinders were soaked in 10% HCl for 24 h, washed with distilled water, sealed, and transported to the sampling point. During sampling, a small amount of ethylene glycol was added to the bottom of the tank to keep the bottom of the tank moist, inhibit the production of microorganisms and algae, and prevent collected dust from leaving the tank. After sample collection, we removed the litter, leaves, insects, and other foreign objects from the dust reduction tank, rewashed the tank wall repeatedly with distilled water, transferred all the liquid in the tank to a polyethylene plastic bottle, sealed it, and brought it back to the laboratory for analysis. The collected dust fall samples were transferred to a beaker, evaporated on a hot plate, and concentrated to 10–20 ml. After cooling, the wall of the cup was rinsed with distilled water and transferred to a porcelain crucible. The evaporate was dried again on a hot plate, placed in an oven at 105 °C for 2 h, and its mass was measured at a repeatedly constant weight (Liu et al., 2019; Wang et al., 2021).

Heavy metal detection requires a microwave digestion/extraction system (ICP-MS, MELSTONE) for digestion and an inductively coupled plasma mass spectrometer (NexION 350X; Perkin Elmer, Waltham, MA, USA) to determine Cu, Cd, Cr, Pb, Zn, Mn, and Co content. Three sets of parallel tests were performed, and the average value was used to determine the concentration of heavy metals. The Chinese National Standard Soil Standard Value (GSS-8) was used for quality control and the recovery rate was calculated to improve the accuracy and precision of the analysis. Concurrently, 10% of the samples were reserved for repeatability testing. The test precision of each heavy metal element was within approximately 5%, the relative double difference was approximately 10%, and the analytical pass rate was 100%. Through random inspection and abnormal point inspection, the results were found to meet the quality control requirements.

## Evaluation method of pollution index

### Nemeiro pollution index

Data were collected as previously described by Xu et al. (2020). Specifically, we used the soil background value that CNEMC (China National Environmental Monitoring Center), (1990) used as the basis for evaluation, as well as the Nemerow pollution index ( ${P_N}$) (Cheng, Zhou & Zhu, 2007), potential ecological risk index ( $RI$) (Zhu, Bian & Li, 2008), geo-accumulation index ( ${I_{geo}}$) (Li et al., 2014), and integrated pollution index (IPI) of dust heavy metals (IPI.dhm). The comprehensive pollution levels of heavy metals in the area’s dust fall were evaluated and analysed (Table 1):

**Table 1:** Classification of pollution index.

Single factor index	Nemerow index	Potential ecological risk index	Enrichment factor	Geo-accumulation index
${P_i}$ ≤ 1 No pollution	${P_N}$ ≤ 1 No pollution	$RI \le \;$50 Minimal ecological risk	$EF{\rm \; } <$ 2 Deficiency to minimal enrichment	${I_{geo}} \le$ 0 No pollution
1 < ${P_i}$ ≤ 2 Slight pollution	1 < ${P_N}$ ≤ 2 Slight pollution	50 $< RI \le \;$100 Medium ecological risk	2 $< \; EF{\rm \; } <$ 5 Moderate enrichment	0 < ${I_{geo}}$ ≤ 1 Slight pollution
2 < ${P_{i\; }}$ ≤ 3 Moderate pollution	2 < ${P_{N\; }}$≤ 3 Moderate pollution	100 $\; < RI \le \;$200 Strong ecological risk	5 $< \; EF{\rm \; } <$ 20 Significant enrichment	1 < ${I_{geo}}$ ≤ 2 Light pollution
${P_i}$ > 3 Heavy pollution	${P_N}$ > 3 Heavy pollution	200 $>$ $RI$ Very strong ecological risk	20 $< \; EF{\rm \; } <$ 40 Very high enrichment	2 < ${I_{geo}}$ ≤ 3 Moderately polluted
			$EF\; >$ 40 Extremely high enrichment	3 < ${I_{geo}}$ ≤ 4 Heavy pollution
				4 < ${I_{geo}}$ ≤ 5 Serious pollution
				5 > ${I_{geo}}$ Severe pollution

(1) $$\matrix{ { {P_i} = {C_i}/{S_i}} \cr }$$

(2) $$\matrix{ { {P_N} = \sqrt {\displaystyle{{{{\left( {\overline {{P_i}} } \right)}^2} + {{\left( {{P_{i{\rm max}}}} \right)}^2}} \over 2}} } \cr }$$

where ${P_i}$ is the pollution index of heavy metal *i* in the soil, ${C_i}$ is the observed concentration of heavy metal $i$ (mg·kg^−1^), and ${S_i}$ is the background concentration of heavy metal $i$ (mg·kg^−1^). The Ningxia environmental soil background value was taken as the soil background concentration of heavy metal *i* China National Environmental Monitoring Center (CNEMC (China National Environmental Monitoring Center), 1990). $\; {P_N}$ is the comprehensive pollution index for soil heavy metals, $\overline {{P_i}}$ is the average number of single factor pollution indices, and ${P_{i{\rm max}}}$ is the largest single factor pollution index.

### Potential ecological risk index

(3) $$\matrix{ {RI = \mathop \sum \limits_{i = 1}^n \; E_r^i = \mathop \sum \limits_{i = 1}^n \; T_r^i \times C_r^i = \mathop \sum \limits_{i = 1}^n {C_i}/C_0^i} \cr }$$

In the formula, $RI$ is the comprehensive potential ecological risk index, $\; \; E_r^i$ is the single potential ecological risk index of heavy metals, and $T_r^i$ is the toxicity parameter of heavy metals, which was determined by Hakanson (1980) after processing the range of toxicity response coefficients: Pb = Cu = Co = 5, Zn = Mn = 1, Cd = 30, Cr = 2. $C_r^i$ is the pollution index of heavy metals, ${C_i}$ is the measured value of heavy metals, and $C_0^i$ is the reference value of heavy metals. This study used the background value of Ningxia province reported by China National Environmental Monitoring Center (CNEMC (China National Environmental Monitoring Center), 1990).

### Geological accumulation pollution index

(4) $$\matrix{ {{I_{geo}} = {\rm lo}{{\rm g}_2}\left( {{C_n}/1.5{B_n}} \right) } \cr }$$

where $\; {C_n}$ is the heavy metal content in dust fall, ${B_n}$ is the content of the soil background value (Ningxia soil background value), and 1.5 is the correction coefficient.

### Comprehensive heavy metal pollution index

Based on the comprehensive pollution status of atmospheric dust fall calculation methods reported by Wu et al. (2020) and Xiong et al. (2016), the IPI of the dust fall heavy metals (IPI.dhm) in the study area was calculated using the following equation:

(5) $$\rm \matrix{ {IPI.\; dhm = {\rm P}{{\rm I}_1} + {\rm P}{{\rm I}_2} + {\rm P}{{\rm I}_3} + {\rm P}{{\rm I}_4}} \cr }$$

where ${\rm P}{{\rm I}_i}$ is the normalised coefficient, ${\rm P}{{\rm I}_1}$ is the normalised coefficient of the single-factor pollution index of dust fall heavy metals, ${\rm P}{{\rm I}_1}\;$ > 0.15, indicates that social and economic activities significantly impact dust fall heavy metals, and ${\rm P}{{\rm I}_2}$ is the normalised coefficient of the background ratio of dust fall heavy metals referenced by the China Soil Environmental Quality Standard (GB 15618-2018). Class II was the evaluation standard. ${\rm P}{{\rm I}_2}$ > 0.15 indicates that the heavy metals in the dust fall contributed significantly to the pollution. ${\rm P}{{\rm I}_3}$ is the normalised coefficient of the dust fall heavy metal geological accumulation index ${I_{geo}}$; ${\rm P}{{\rm I}_3}\;$ > 0.15 indicates that pollution is a significant factor in dust fall heavy metals. ${\rm P}{{\rm I}_4}$ is the normalised index of the potential risk index of dust fall heavy metals, and heavy metals with ${\rm P}{{\rm I}_4}\;$ > 0.15 were considered significant pollution factors in dust fall. ${\rm IPI}.{\rm dhm}$ is the comprehensive pollution index of heavy metals in dust fall and a ${\rm IPI}.{\rm dhm}$ > 0.5 is considered a significant pollution factor.

### Enrichment factor (EF) method

The heavy metal EFs were calculated using the following equation:

(6) $$\matrix{ {EF = {{\left( {\displaystyle{{{C_i}} \over {{C_n}}}} \right)}_{sample}}/{{\left( {\displaystyle{{{C_i}} \over {{C_n}}}} \right)}_{background}}} \cr }$$

where ( $\displaystyle{{{C_i}} \over {{C_n}}}$) is the concentration ratio between the heavy metal and the reference element in both the sample and the background soil of Ningxia province reported by the China Environmental Monitoring Center (CNEMC (China National Environmental Monitoring Center), 1990). Mn was chosen as the reference in this study as it is a conserved element. Five contamination categories were recognized on the basis of the EF (Table 1; Lu et al., 2009).

### Positive matrix factorization model (PMF)

The PMF model was developed by Paatero & Tapper (1994). The United States Environmental Protection Agency’s (EPA) PMF is one of the receptor models developed by their Office of Research and Development (ORD). Its greatest advantage is that it requires no source profiles and uses uncertainty to weigh all data. The goal of the PMF is to determine source profiles and source contributions based on a composition dataset (Norris et al., 2014):

(7) $$\matrix{ {{{\rm X}_{ij}} = \mathop \sum \limits_{K = 1}^P \left( {{G_{ik}}{F_{kj}} + {E_{ij}}} \right)} \cr }$$

where ${{\rm X}_{ij}}$ is the concentration of species $j$ in sample $i$, $p$ is the number of factors, ${G_{ik}}$ is the contribution of factor $k$ in sample $i$, ${F_{kj}}$ is the concentration of species $j$ in factor $k$, and ${E_{ij}}$ is the error of the species $j$ in sample $i$.

Factor profiles and contributions are derived using a PMF model that minimizes the objective function Q:

(8) $$\matrix{ { Q = \mathop \sum \limits_{i = 1}^n \mathop \sum \limits_{j = 1}^m {{\left[ {\displaystyle{{{X_{ij}} - \mathop \sum \limits_{k = 1}^p {g_{ik}}{f_{kj}}} \over {{u_{ij}}}}} \right]}^2}} \cr }$$

where $Q$ is the cumulative residual, $i$ is the number of samples, $j$ is the type of measured pollutants, $p$ is the number of suitable factors, $f$ is the component matrix of each source, $g$ is the contribution matrix of each pollutant in the sample, and ${u_{ij}}$ is the uncertainty of the types of contaminants in the sample. The calculation formula is as follows:

(9) $$\matrix{ {{u_{ij}} = \left\{ {\matrix{ \displaystyle{5 \over 6} \times MDL (c \le MDL) \cr \sqrt {\left( {{{\left( {error\; fraction\; \times c} \right)}^2} + MD{L^2}} \right)} (c \gt MDL)} \cr } \right.} \cr }$$

where $c$ is the measured content and $MDL$ is the method’s detection limit.

## Health risk assessment method

### Exposure risk

Drawing from the U.S. EPA’s risk model and previous studies on heavy metals in industrial areas, we revised some parameters (Yang et al., 2016). Since the study area is in a main area of industrial activity, the exposure parameters should only consider adult values, parameters, and reference values in domestic and foreign contexts (DB11/T 656-2009, 2009; United States. Environmental Protection Agency. Office of Wastewater Management, 1995; Yang et al., 2016). Eqs. (10)–(12) were used to evaluate three heavy metals in hand-to-mouth, inhalation, and skin contact dust exposure pathways:

(10) $$\matrix{ {AD{D_{ing}} = C \times \displaystyle{{EF \times ED} \over {AT \times BW}} \times IngR \times {{10}^{ - 6}}} \cr }$$

(11) $$\matrix{ {AD{D_{inh}} = C \times \displaystyle{{EF \times ED} \over {PEF \times AT \times BW}} \times InhR \times {{10}^{ - 6}}} \cr }$$

(12) $$\matrix{ {AD{D_{derm}} = C \times \displaystyle{{EF \times ED} \over {AT \times BW}} \times SL \times SA \times ABS \times {{10}^{ - 6}}} \cr }$$

where $AD{D_{ing}}$, $AD{D_{inh}}$, and $AD{D_{derm}}$ are the daily average intake of heavy metals from hand-to-mouth exposure, inhalation, and skin contact, mg/(kg∙d); $C$ is the measured concentration of heavy metals in dust fall in the district, mg/kg; $EF$ is the exposure frequency, 300 d/a; $ED$ is the exposure period, 30 a; $AT$ is the average exposure time; the non-carcinogenic effect is $ED$ × 365 d; and the carcinogenic effect is 70 (lifetime) × 365 d; $BW$ is the average weight, 60 kg; $IngR$ is the intake dust reduction rate, 50 mg/d; $InhR$ is the respiration rate, 13.77 m^3^/d; $PEF$ is the particulate matter emission factor, 1.36 × 109 m^3^/kg; $SL$ is skin adhesion, 0.07 mg/cm^2^; $SA$ is the exposed skin area, 4,350 cm^2^/d; and ABS is the skin absorption factor, 0.001 dimensionless.

### Risk characterization

Heavy metal elements Cd, Cu, Pb, Cr, Zn, Mn, and Co can be dangerous to human health. Among these, Cd and Pb have both non-carcinogenic and carcinogenic risks (Xing et al., 2010; Fang et al., 2015), according to relevant domestic and foreign research, related guidelines (DB11/T 656-2009, 2009; United States. Environmental Protection Agency. Office of Wastewater Management, 1995; Yang et al., 2016; Shahab et al., 2020), and the risk reference dose (RfD; Table 2). The calculation formulae are as follows:

**Table 2:** Reference measurement of heavy metal elements in dust *via* three exposure routes.

Item	Cu	Cd	Cr	Pb	Zn	Mn	Co
$Rf{D_{ing}}$	4.00 × 10^−2^	1.00 × 10^−3^	3.00 × 10^−2^	3.50 × 10^−3^	3.00 × 10^−1^	4.60 × 10^−2^	2.00 × 10^−2^
$Rf{D_{inh}}$	4.02 × 10^−2^	1.00 × 10^−3^	4.02 × 10^−2^	3.52 × 10^−3^	3.00 × 10^−1^	1.43 × 10^−5^	5.71 × 10^−6^
$Rf{D_{derm}}$	1.2 × 10^−2^	5.00 × 10^−5^	1.20 × 10^−2^	5.25 × 10^−4^	6.00 × 10^−2^	1.84 × 10^−3^	1.60 × 10^−2^
$SF$		8.4		8.5 × 10^−3^

(13) $$\; \matrix{ { H{Q_{ij}} = \displaystyle{{AD{D_{ij}}} \over {Rf{D_{ij}}}}}}$$

(14) $$\matrix{ { HI = \mathop \sum \limits_{i = 7}^{j = 3} H{Q_{ij}}} \cr }$$

(15) $$\matrix{ { CR = \mathop \sum \limits_{i = 3}^{j = 3} AD{D_{ij}} \times S{F_{ij}}} \cr }$$

(16) $$\matrix{ {TCR = \mathop \sum \limits_{i = 2}^{j = 3} C{R_{ij}}} \cr }$$

where $AD{D_{ij}}$ is the exposure dose mg/kg·d of $j$ exposure route of heavy metal element $i,\; Rf{D_{ij}}$ is the reference dose mg/kg·d of the $j$ exposure route of heavy metal element $i$, and $SF$ slope factor is the carcinogenic factor of certain metal pollutants. $H{Q_{ij}}$ is the risk quotient of non-carcinogenic heavy metals $i$ under the $j$ exposure route, $HI$ is the total non-carcinogenic risk index of seven heavy metals under the three exposure routes, $C{R_{ij}}$ is the risk index of the $j$ exposure route of carcinogenic heavy metal $i$, $S{F_{ij}}$ is the carcinogenic slope of the $j$ exposure route of pollutant $i$ kg·d/mg, ${\rm and}\ TCR$ is the total carcinogenic risk index of two carcinogenic heavy metals under three exposure routes.

### Data analysis

We used IBM SPSS STATISTICS 22.0 and Microsoft Office Excel 2019 for dust descriptive statistical analysis and correlation analysis of heavy metal content. We also used Origin 9.0 to make box plots and ArcGIS10.2 kriging interpolation to determine the spatial distribution of heavy metal concentrations. Finally, we used EPA PMF 5.0 for pollution source analysis.

## Analysis and discussion

### Heavy metal content in dust reduction

The statistical results for the heavy metals in the area’s dust fall are shown in Table 3. The average concentrations of Cu, Cd, Cr, Pb, Zn, Mn, and Co were 20.00 ± 5.74, 0.39 ± 0.24, 39.84 ± 8.76, 91.55 ± 25.57, 222.82 ± 159.40, 584.31 ± 89.16, and 8.49 ± 1.87 mg·kg^−1^, respectively. Heavy metal concentration for all seven elements were quite different in the dust fall. However, all elements were lower than the soil pollution risk screening value found in the ‘Soil Environmental Quality Agricultural Land Soil Pollution Risk Control Standard’ (GB 15618-2018II). Based on the soil background value in Ningxia, six elements (all but Cr, which did not exceed the standard) exceeded limits by the following rates: Cu, 62.16%; Cd, 94.59%; Pb, 100%; Zn, 91.89%; Mn, 24.32% and Co, 2.71 %. Among these, Cd, Pb, and Zn exceeded the thresholds by the highest amounts and accumulated to varying degrees in the study area. These findings are consistent with our previous researching indicating that there are over-standard heavy metal rates in the study area’s soil (Xu et al., 2020). According to Wilding’s (1985) classification of the degree of variation, Cu, Cr, Pb, Mn, and Co (0.287, 0.220, 0.301, 0.153, and 0.221) were moderately variable (0.15 < CV < 0.36). The coefficients of variation of Zn and Cd were 0.715 and 0.605, respectively, and were highly variable (CV > 0.36). This indicates that the overall distribution of these two elements’ concentrations was relatively discrete, and there may be areas with more serious pollution. Wu et al. (2020) conducted a study in the coal mining area of Wuhai City, Inner Mongolia, and found that the Cd and Zn levels outside of the mine were significantly higher than in other areas, which was related to coal transportation. In this study, the region’s industrial parks mainly produce coal chemicals. Therefore, we speculated that the variations in Zn and Cd levels may be affected by industrial park activity.

**Table 3:** Descriptive statistics of heavy metals in dust fall in the study area.

Quality index	Max	Min	Mean	Standard deviation	CV	Background values^a^	GB 15618-2018II pH > 7.5/mg·kg−1
Cu (mg·kg^−1^)	30.39	9.86	20.00	5.74	0.287	22.1	100
Cd (mg·kg^−1^)	1.04	0.09	0.39	0.24	0.605	0.11	0.6
Cr (mg·kg^−1^)	55.79	21.59	39.84	8.76	0.220	60.6	250
Pb (mg·kg^−1^)	167.73	60.13	91.55	27.57	0.301	20.6	200
Zn (mg·kg^−1^)	747.02	41.63	222.82	159.40	0.715	58.8	300
Mn (mg·kg^−1)^	673.38	325.33	584.31	89.16	0.153	524	1,200
Co (mg·kg^−1)^	12.87	5.54	8.49	1.87	0.221	12.6	24

**Notes:**

Soil background^a^ (China National Environmental Monitoring Centre).

GB 15618-2018II: ‘Soil Environmental Quality Agricultural Land Soil Pollution Risk Control Standards’ screening value of soil pollution Risk State Environmental Protection Administration of China GB 15618-2018II.

### Evaluation of heavy metal pollution characteristics in dust fall

**Figure 2:**
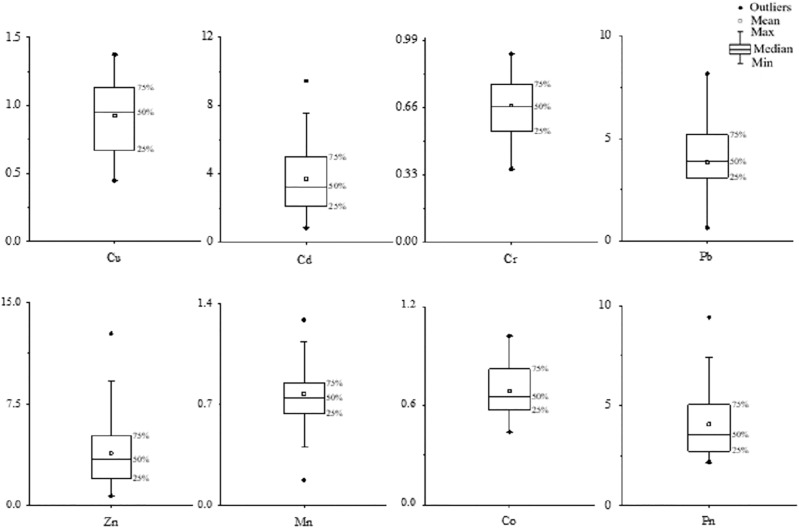
Single factor and Nemeiro pollution index box plot.

We evaluated the overall study area’s pollution using Eq. (5). As shown in Table 4, the single dust heavy metal pollution index normalization coefficient ( ${\rm P}{{\rm I}_1}$) Zn > Pb > Cd > Cu > Mn > Co > Cr. $SPI$ stands for the single factor pollution index of heavy metals in dust fall. According to the classification basis of the single factor pollution index in Table 1, the box diagram of the combined pollution index (Fig. 2) shows that Cu, Mn, and Co were slightly polluting; Cr was non-polluting; Zn, Pb, and Cd were severely polluting. Clear local aggregation and point source pollution trends were observed. The Nemeiro pollution index ${P_N}$ was 4.04, indicating that the study area was heavily polluted. The Nemeiro pollution index formula emphasises the characteristics of the most polluting elements. The Pb and Zn elements’ ${\rm P}{{\rm I}_1}$ was greater than 0.5 in the desert dust fall, indicating that it was most affected by social and economic activities. The two elements have a strong influence. ${\rm P}{{\rm I}_2}$ is the normalised coefficient of the background ratio of dust fall heavy metals. $BR$ stands for the ratio between the average value of dust fall heavy metal content and the soil background value of the corresponding heavy metal. Zn is the element with the highest contribution rate of pollution in dust fall, and Cd, Pb, Mn, and Co were significant contributors to pollution ( ${\rm P}{{\rm I}_2}\;$ > 0.15).

**Table 4:** Comprehensive pollution index of heavy metals in dust fall in the study area.

Element	mg/kg	$SPI$	${\rm P}{{\rm I}_1}$	$BR$	${\rm P}{{\rm I}_2}$	${I_{geo}}$	${\rm P}{{\rm I}_3}$	$Er$	${\rm P}{{\rm I}_4}$	$IPI\; {\rm d}hm$	$EF$
Cu	20.27	0.93	0.04	0.20	0.04	−0.77	0.17	4.52	0.04	0.28	1.13
Cd	0.40	3.67	0.5	0.67	0.44	1.04	0.83	106.31	1	2.77	4.18
Cr	40.11	0.66	0	0.16	0	−1.22	0	1.32	0.01	0.01	0.80
Pb	91.55	4.44	0.63	0.46	0.26	1.51	1	22.22	0.20	2.09	5.39
Zn	393.86	6.70	1	1.31	1	1.22	0.89	3.79	0.03	2.92	6.71
Mn	441.47	0.84	0.03	0.37	0.18	−0.88	0.12	0.84	0	0.33	–
Co	8.62	0.68	0.004	0.36	0.17	−1.16	0.02	3.37	0.02	0.22	0.83

**Figure 3:**
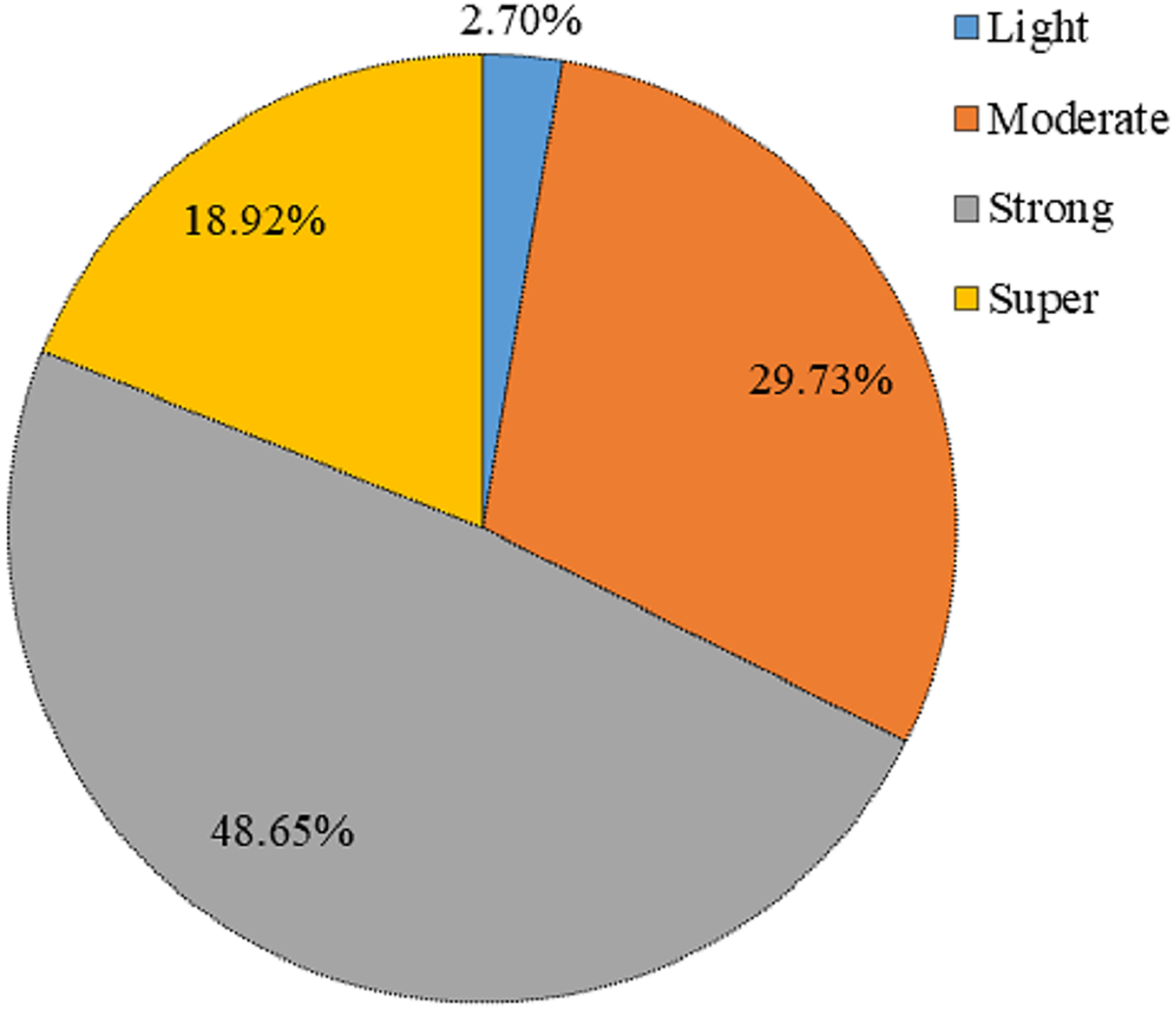
Pie chart showing percentages of potential heavy metal ecological hazard in the study area.

The geo-accumulation index fully considers the differences in background values in different regions and is widely used to assess soil and dust heavy metal pollution (Muller, 1969). Its characteristics can reflect natural changes in the distribution of heavy metals and the impact of human activities on the environment. This is an important parameter for determining the effects of human activities. ${\rm P}{{\rm I}_3}$ is the normalisation coefficient of the geological accumulation index of dust fall heavy metals ${I_{geo}}$. The ${\rm P}{{\rm I}_3}$ of Zn, Pb, and Cd were all greater than 0.5, indicating that the degree of pollution was extremely high. Cd ( ${\rm P}{{\rm I}_4}$ > 0.5) in the dust fall of the desert steppe showed a very high ecological risk. According to Fig. 3, only 2.7% of the study area was at a slight ecological risk, 29.73% was at a medium ecological risk, and most samples were at a strong ecological risk. 18.92% of the sample plots showed serious pollution, and Cd was the root cause of this phenomenon; this is because Cd’s toxicity parameter is relatively large. Zhang, Shao & Wei (2019) found that that the Cd content in the dust fall of Kaifeng City was highly polluted, and the potential ecological hazard index of Cd was high, especially in industrial and residential areas. Of course, some studies have found that Cd pollution in dust fall is more a serious and common problem (Han et al., 2021), especially along highways and in other areas (Faiz et al., 2009). Cd’s large toxicity parameter means that it causes greater harm to the environment (Xu et al., 2008). Cd’s over-standard rate in the study area was 94.59%, indicating a high ecological risk index.

The ranking order of IPI.dhm values was Zn > Cd > Pb > Mn > Cu > Co > Cr. Four pollution index evaluation methods were integrated. The comprehensive pollution indices of Zn, Cd, and Pb in the dust fall of the desert steppe were 2.922, 2.771, and 2.087, respectively (IPI.dhm > 0.5). These three heavy metal elements were affected by the combination of social and economic activities and other human factors, which all significantly influence dust fall in the study area. After referencing previous research and our calculations, we choose Mn as the reference factor (Lu et al., 2009). The order of $EF$ of dustfall heavy metal elements in the study area was Zn > Pb > Cd > Cu > Co > Cr, where Pb and Zn were significant pollution factors ( $EF$ > 5), which was consistent with the previous analysis results.

## Source analysis of heavy metals in dust fall

### Correlation analysis

Correlation analysis is useful for identifying the sources of heavy metals (Lv et al., 2013). A significant correlation indicates strong homology, concomitance, and a strong relation between elements that may be from similar sources or exhibit compound pollution, while a weak correlation may indicate a difference in sources (Zheng et al., 2013). Table 5 shows that the correlation coefficients among elements in the atmospheric dust fall of the desert steppe (Cu−Cd (0.803), Cu–Pb (0.853), Cu–Co (0.717), Cd–Pb (0.841), Cd–Mn (0.795), the correlation coefficients of Co-Cd (0.828), Cr–Mn (0.870), Cr–Co (0.705), and Cd–Mn (0.795)) were all greater than 0.7, indicating a very significant correlation (*P* < 0.01). According to the correlation coefficient, we speculate that Cu–Cd–Pb–Co has a composite source and Cr–Mn has a similar source. As the correlation coefficient between Zn and the other six elements was low, we determined that it had a separate source of pollution.

**Table 5:** Correlation analysis of heavy metals in dust fall in the study area.

Element	Cu	Cd	Cr	Pb	Zn	Mn	Co
Cu	1						
Cd	0.80**	1					
Cr	0.27	0.55**	1				
Pb	0.85**	0.84**	0.45**	1			
Zn	0.33*	0.41*	0.38*	0.31	1		
Mn	0.56**	0.80**	0.87**	0.69**	0.46**	1	
Co	0.72**	0.83**	0.71**	0.80**	0.43**	0.84**	1

**Note:**

**indicates *P* < 0.01, the correlation is extremely significant; *indicates *P* < 0.05, and the correlation is significant.

### Positive matrix factorization model analysis

**Figure 4:**
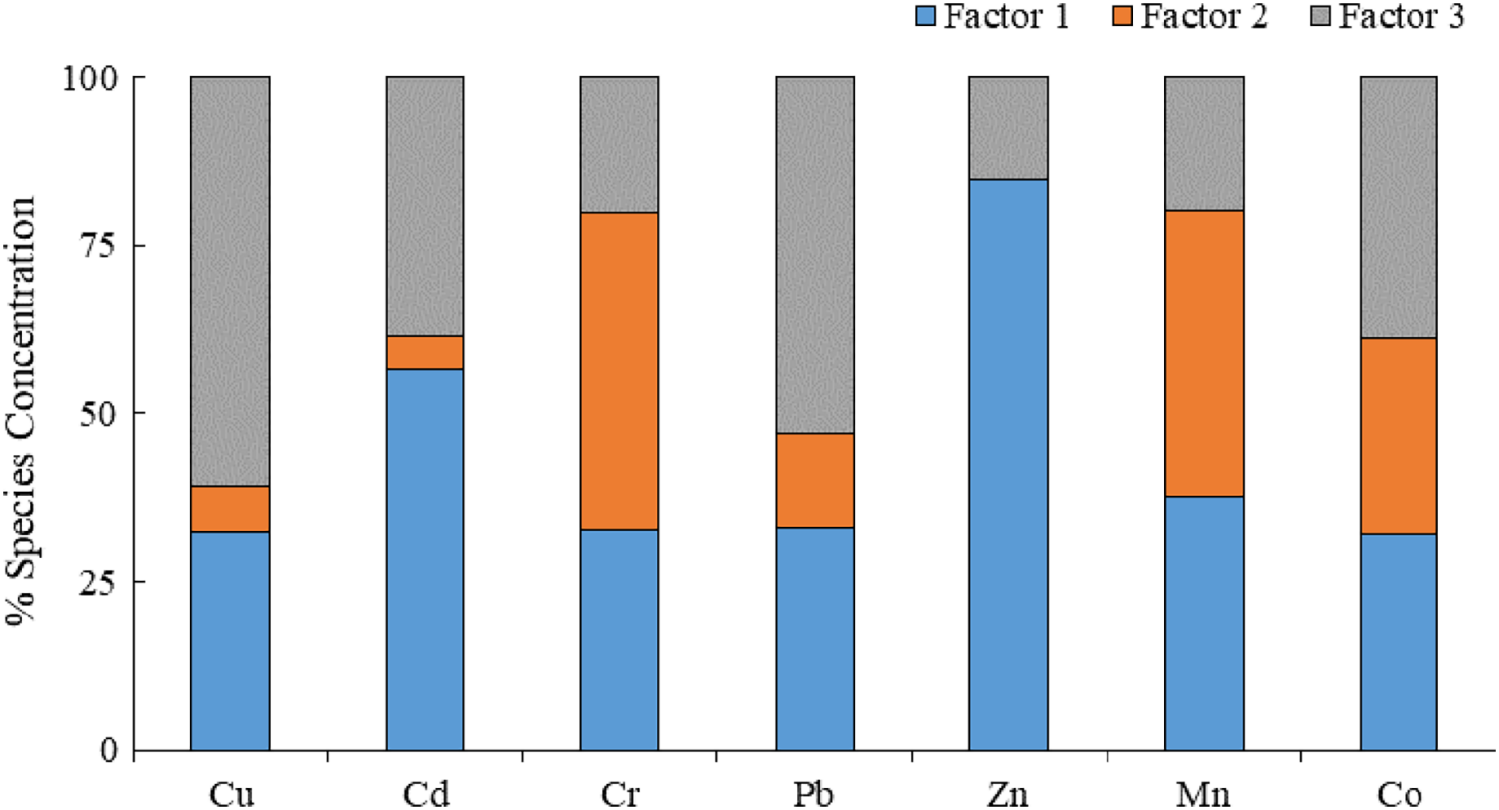
Source analysis of heavy metals based on the PMF model.

**Figure 5:**
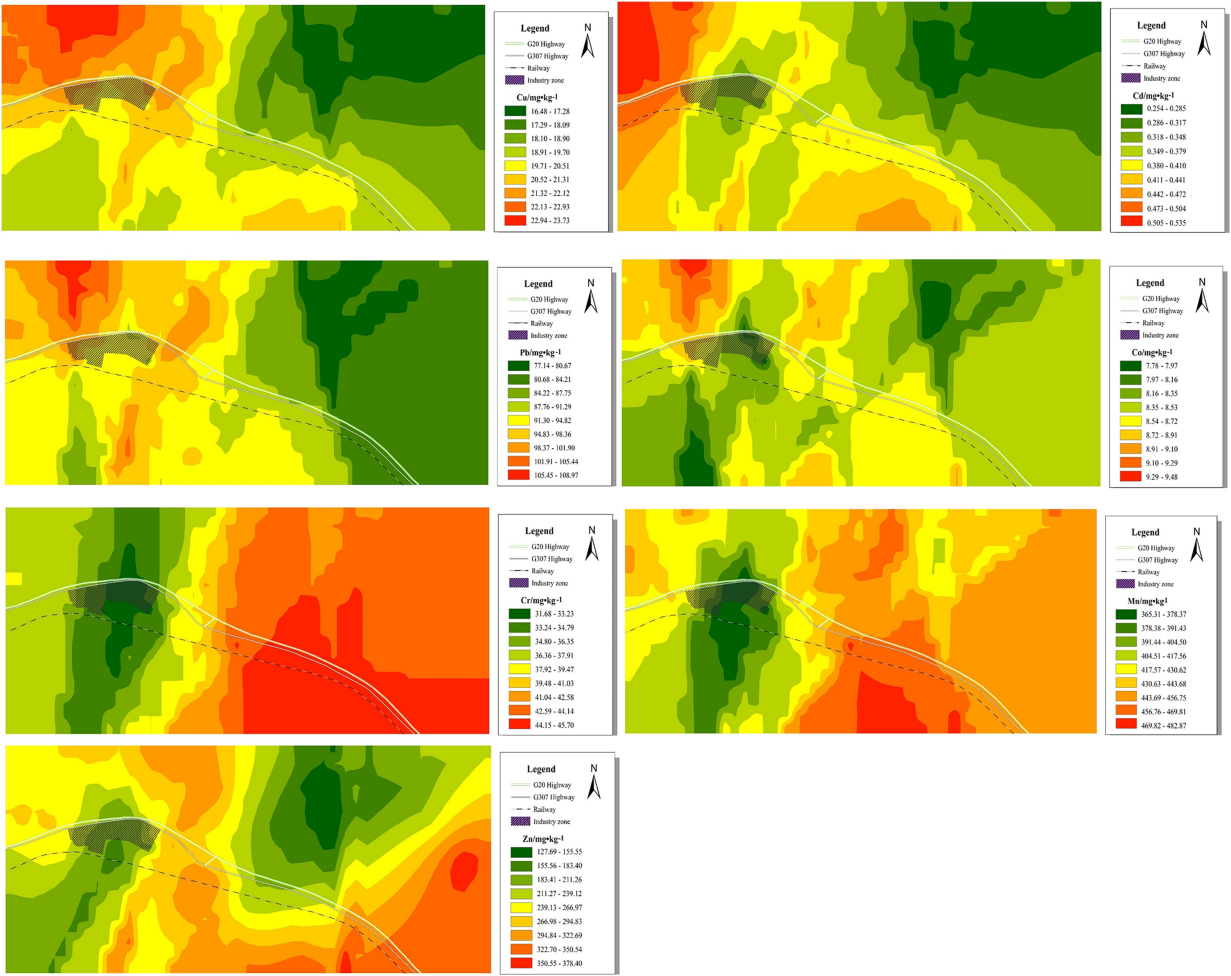
Spatial distribution of heavy metals in the area’s dust fall.

Our findings were determined using the EPA PMF5.0 software with the number of words set to three. The model was iterated at least 77 times and the residual was between −3 and 3. The determination coefficients of the seven elements were as follows: Cu (0.95), Cd (0.68), Cr (0.80), Pb (0.93), Zn (0.99), Mn (0.99), and Co (0.88). The determination coefficients of all heavy metals were higher than 0.6, indicating that the PMF model had a good fitting effect (Chai et al., 2021). According to Fig. 4, factor 1 accounted for 44.08% of the total source. Zn and Cd had higher concentrations on factor 1, and the contribution rate of Zn was 84.67%. Studies on atmospheric dust fall reported that Zn likely comes from the combustion of engines and fossil fuels (Monaci et al., 2000; Duong & Lee, 2011). Additionally, some researchers have found that Zn in dust is usually related to tire wear emission sources (Councell et al., 2004; Pant & Harrison, 2013; Valotto et al., 2015; Valotto, Cattaruzza & Bardelli, 2017). Hwang et al. (2016) found that high Zn concentrations were mainly due to tire wear and natural factors. Zn concentration is mainly affected by transportation. In the study area, the coefficients of variation and over-standard rates of Zn were relatively high, and the distribution was relatively discrete. The average single-factor pollution index of Zn was 4.07, which is a significant pollution factor among the seven dust-reducing heavy metal elements and indicates heavy pollution. Combined with the spatial distribution map (Fig. 5), Zn was shown to be highly diffusible. In addition to accumulating along both sides of the highway, we also observed a small-scale enrichment phenomenon in the northern part of the industrial park. With the continuous promotion of new energy electric vehicles and the prohibition of unleaded gasoline, automobiles have relatively high weights, which increases emissions from typically non-emission sources such as brakes, tires, and asphalt wear (Timmers & Achten, 2016). Zn is the main metal component released by car tire wear (Hwang et al., 2016). In the study area, G307 and G20 are important local transportation arteries with large annual traffic volumes. To reduce transportation costs, some truck drivers have overloaded cargo, and wind-borne dust can easily cause superimposed pollution. In summary, factor 1 is a source of traffic pollution.

Factor 2 accounted for 20.60% of the total source. The contribution rates of Cr and Mn in factor 2 were 46.99% and 42.47%, respectively. Studies have shown that Cr is the main source of industrial pollution (Guan et al., 2018), and Mn is an indispensable metal element for the deep processing of aluminium products and alloys (Zhang et al., 2016). The two elements mostly come from steel plants and traffic emissions (Wang et al., 2018) and easily accumulate on both sides of transportation routes (Hwang et al., 2016; Shi et al., 2008; Zgłobicki, Telecka & Skupiński, 2019). Research has also found that climate change can affect the composition and content of heavy metal dust (Wang et al., 2017). In different dust storm events, the heavy metal content in dust varies with wind speed. During periods of minimal rain and weather, the atmospheric boundary layer gradually decreases, thereby increasing the suspension of particulate matter in the air. In a study of dust fall in arid and semi-arid areas in northern China, Zhuang et al. (2001) found that heavy metal elements, such as Cr and Mn, make up the earth’s crust. In this study, the coefficient of variation of Cr and Mn was small, the individual pollution index was 0.66 and 0.46, and the comprehensive pollution index was low. The low $EF$ of Cr suggest a crustal influenced. Combined with the current situation in the study area, Cr and Mn can be identified as natural resources. Heavy metal elements in dust are more susceptible to the influence of complex topography, landscape features, wind direction, and human factors, resulting in a different diffusion of pollutants in the atmosphere (Aerzuna et al., 2017). Figure 5 shows that Cr and Mn have similar spatial distributions and are affected by the dominant wind, northwest wind, topography of the high south and low north, and high values that converge in the southeast region as non-point source pollution. The study area, which is the main area for industrial production and raw material transportation, is crossed by the Qingyin Expressway, 307 National Road, and the Taiyin Railway. During transportation, there is an insufficient adsorption capacity of heavy metals in dust. Throughout the year, the study area is subject to heavy wind and sand, exposed surface water and soil, and loose soil particles. Organic matter and nutrient contents are also low, and the desert steppe cannot effectively block strong winds. In arid climates, sand and dust are widespread because of factors such as air erosion or vibration. In addition, Highway with transportation also resuspended the road dust. Therefore, factor 2 is a source of natural factors.

Factor 3 accounted for 35.32% of the total source. Cu, Pb, and Co had higher concentrations in factor 3, and their contribution rates were 61.05%, 53.07%, and 38.92%, respectively. It is generally believed that Cu and Pb are related to human transportation (Al-Khashman, 2004; Sezgin et al., 2004; Han et al., 2007). High concentrations of Cu and Pb accumulate along roads (Guan et al., 2019) because Pb is a main component in tires and wheels (Hwang et al., 2016) and the wear of brakes and tires releases metal elements such as Cu and Pb (Hjortenkrans, Bergbäck & Häggerud, 2006). Co concentrations generally result from the use of chemical reagents (Zhang et al., 2015). Higher Co concentrations have arisen from coal combustion and metallurgical production (Li & Feng, 2010), and lower concentrations have been found in the soil parent material (Bai et al., 2014). Combined with the spatial distribution map (Fig. 5), the continuous spatial distribution of variables was obtained by interpolating data from discrete points. Higher Cu, Pb, and Co concentrations were found in the northwest area of the industrial park, and the southeast was a low-value area. There is a strong correlation between heavy metals and industrial activities in relatively densely populated areas. During the sample collection period, a photovoltaic solar power station was built in the northwest of the study area. Influenced by climate and other factors, a local concentration was formed during construction and the point source pollution was distributed in space. The synergistic influence of the dominant wind direction and adjacent industrial parks was the key factor increasing the region’s heavy metal content (Fan et al., 2013). Cu, Pb, and Co display noticeable enrichment characteristics in industrial parks and transportation routes, and they generally decrease from northwest to southeast. In the correlation analysis, these elements had a strong positive correlation with each other (Table 5). We speculated that factor 3 was affected by the synergistic influence of a variety of human factors, making it a source of compound pollution. According to this analysis, factor 1 > factor 3 > factor 2.

## Health risk assessment of dust falling heavy metals

### Average daily exposure to heavy metals in dust fall

As shown in Table 6, the daily average exposure to non-carcinogenic risks was Mn > Zn > Pb > Cr > Cu > Co > Cd, which was consistent with the measured concentration of heavy metals in the study area’s atmospheric dust fall. The study area was located in a desert steppe zone with many factories, and the population most potentially affected was adult men. Therefore, the impact of heavy metals in dust fall on children was not considered in this experiment. The average daily exposure doses of heavy metals in dust reduction across different exposure routes in the study area were hand-to-mouth > inhalation > skin contact, and accounted for 65.57%, 34.03%, and 0.399% of the total daily average exposure, respectively. The hand-to-mouth route was the most prevalent exposure route in the study area. Windy weather often occurs in desert steppe areas, which undoubtedly increases the risk of dust intake in conjunction with frequent industrial activities. Dust particles, especially fine particles, from heavy metals can be resuspended in the atmosphere, thus increasing exposure risk (Mohmand et al., 2015; Zheng et al., 2010).

**Table 6:** Average daily does for heavy metals and exposure routes in study area.

Risk exposure	Element	$AD{D_{ing}}$	$AD{D_{inh}}$	$AD{D_{derm}}$
Non-carcinogenic risk exposure	Cu	1.39E−05	7.20E−06	8.45E−08
	Cd	2.77E−07	1.44E−07	1.68E−09
	Cr	2.75E−05	1.43E−05	1.67E−07
	Pb	6.27E−05	3.26E−05	3.82E−07
	Zn	2.70E−04	1.40E−04	1.64E−06
	Mn	3.02E−04	1.57E−04	1.84E−06
	Co	5.90E−06	3.06E−06	3.60E−08
Carcinogenic risk exposure	Cd	1.18E−07	1.43E−07	3.62E−08
	Pb	2.69E−05	3.25E−05	7.17E−08

### Non-carcinogenic and carcinogenic health risk assessment of heavy metals in dust reduction

The individual non-carcinogenic risk quotient (HQ) and total non-carcinogenic risk index (HI) of the seven atmospheric dust heavy metals in the study area across evaluated exposure routes were calculated using formulas (13) and (14). The individual carcinogenic risk (CR) and total carcinogenic risk (TCR) values of Pb and Cd in the study area were calculated using formulas (15) and (16). The results are listed in Table 7.

**Table 7:** Impact of dust falling heavy metals under different exposure routes on human health risks.

Types of health risks	Element		Minimum	Maximum	Mean	SD
Non-carcinogenic risk	$H{Q_{ing}}$	Cu	1.72E−04	5.20E−04	3.47E−04	9.38E−05
		Cd	6.49E−05	7.11E−04	2.76E−04	1.59E−04
		Cr	4.93E−04	1.27E−03	9.16E−04	1.98E−04
		Pb	1.18E−02	3.28E−02	1.79E−02	5.40E−03
		Zn	9.51E−05	1.35E−02	8.99E−04	2.17E−03
		Mn	3.15E−03	1.00E−02	6.57E−03	1.68E−03
		Co	1.90E−04	4.41E−04	2.95E−04	6.38E−05
	$H{Q_{inh}}$	Cu	8.91E−05	2.70E−04	1.80E−04	4.87E−05
		Cd	3.37E−05	3.69E−04	1.43E−04	8.26E−05
		Cr	1.91E−04	4.93E−04	3.55E−04	7.68E−05
		Pb	6.07E−03	1.69E−02	9.25E−03	2.79E−03
		Zn	4.93E−05	7.02E−03	4.67E−04	1.13E−03
		Mn	5.27	16.74	10.98	2.80
		Co	3.45E−01	8.01E−01	5.37E−01	1.16E−01
	$H{Q_{derm}}$	Cu	3.49E−06	1.06E−05	7.04E−06	1.90E−06
		Cd	7.90E−06	8.66E−05	3.36E−05	1.94E−05
		Cr	7.50E−06	1.94E−05	1.39E−05	3.02E−06
		Pb	4.78E−04	1.33E−03	7.27E−04	2.19E−04
		Zn	2.89E−06	4.12E−04	2.74E−05	6.61E−05
		Mn	4.80E−04	1.53E−03	1.00E−03	2.55E−04
		Co	1.45E−06	3.36E−06	2.25E−06	4.85E−07
Carcinogenic risk	$HI$		5.71	17.61	11.63	2.91
	$CR$	Cd	6.84E−07	6.12E−06	2.50E−06	1.33E−06
		Pb	3.32E−07	9.26E−07	5.06E−07	1.52E−07
	$TCR$		1.02E−06	7.04E−06	3.01E−06	1.46E−06

The non-carcinogenic risk of dust fall heavy metal elements to the human body across the three exposure routes had HQ rankings of inhalation > hand-to-mouth > skin contact. The heavy metal HQ through hand-to-mouth and skin contact exposure route was less than one, and there was no non-carcinogenic risk. Mn ingested through the respiratory route had a non-carcinogenic risk (HQ: 5.27–11). There are great differences between different heavy metal elements. The individual non-carcinogenic health risk indices of heavy metal elements, except for Mn, through the three exposure routes were all less than one, which was within the safe range and indicated that they would not cause harm to human health. However, the HI is relatively high in the area where Mn accumulated. This phenomenon warrants greater attention. The average value of the total HI was 11.6, indicating a risk to non-carcinogenic health. We concluded that heavy metals in the study area pose a certain threat to human health. The total HI values of the seven heavy metal elements ranked Mn > Co > Pb > Zn > Cr > Cu > Cd, and only Mn had an HI greater than one. Mn was the most important element that created non-carcinogenic hazards in dust reduction. The TCR values of the two carcinogenic heavy metals showed that Cd > Pb, and the carcinogenic index of dust fall heavy metals exposed by the three methods to the human body was less than 10^−6^, indicating no carcinogenic health risk for Cd and Pb in the evaluated pathways. For both non-carcinogenic and carcinogenic risks, the exposure routes were ranked as follows: inhalation > hand-to-mouth > skin contact.

Health risk assessment is very important for quantifying human health when exposed to heavy metals. Although some elements are required by the human body, long-term inhalation exposure can affect the respiratory system and increase the risk of lung and nasal cancers (Ghosh et al., 2018). Currently, the contents of heavy metals and other pollutants released by the burning of fossil fuels, traffic emissions, and the wear and tear of automobile installations are gradually increasing. These pollutants can easily accumulate in the human body due to wind, traffic, and other factors (Duzgoren-Aydin et al., 2006). Studies have reported that long-term exposure to high concentrations of Mn can affect the central nervous system and cause intoxication symptoms such as weakness, lethargy, tremor, and hypoxemia (Sah et al., 2019). Considering the low over-standard rate of Mn in the study area, wearing masks or taking other protective measures when in contact with industrial production and transportation can effectively prevent dust from entering the body. Although Zn, Cd, and Pb were comprehensively significant pollution factors in this study, their low health risk indices indicated that they did not pose a high health risk to adults. The non-carcinogenic and carcinogenic risk indices of the six heavy metals, excluding Mn, were within the acceptable range.

## Conclusion

This study comprehensively analysed the pollution characteristics of heavy metals in the dust fall of the desert steppe, an industrial area, using the Nemeiro pollution, geo-accumulation, and potential ecological risk indices. To further determine the source of pollution, the correlation and PMF model were combined with geostatistical evaluation methods to identify the distribution of heavy metal elements in the area’s dust fall. Considering the carcinogenic risk for residents near the study area, a quantitative assessment was made using the exposure risk and risk characterisation of the health risk model. The main conclusions were as follows:

(1) Using Ningxia’s soil background value as the standard, the study area’s Melo pollution index of 4.04 indicated heavy pollution, and 48.65% of the samples showed strong ecological risks. The over-standard rates of Cu, Cd, Pb, Zn, Mn, and Co were 62.16%, 94.59%, 100%, 91.89%, 24.32%, and 2.71%, respectively (Cr was excluded). The ranking of the comprehensive pollution index (IPI.dhm) was Zn > Cd > Pb > Mn > Cu > Co > Cr. The excess rates of Zn, Cd, and Pb in the study area were above 90%. These three elements were affected by the synergistic influence of social and economic activities and human factors. Zn is the element with the highest contribution rate of pollution in dust fall, and the degree of pollution was very high.

(2) In the correlation and PMF models, Zn and Cd were considered to have traffic pollution sources, and the higher pollution index was concentrated in the northern area of the industrial park and on both sides of the highway. Cr and Mn had influence of natural factors, and high values were concentrated in the southeast region as non-point source pollution. Cu, Pb, and Co were composite pollution sources that were affected by the dominant wind direction and adjacent areas. High values were concentrated in the northwest region of the industrial park, and the southeast region was a low-value area. Based on the multivariate analysis results, Zn, Cd, and Pb were significant pollution factors in the study area. These three elements were all related to the wear of automobile brakes and tires. Although there are many types of industrial activities, the analysis results confirmed that the study area was deserted. Heavy metals in the grassland dust were severely affected by transportation, and their accumulation characteristics were observable around the industrial park and on both sides of the transportation route.

(3) Across different exposure routes, the average daily exposure doses of heavy metals in the study area ranked hand-to-mouth > inhalation > skin contact, and accounted for 65.57%, 34.03%, and 0.399% of the total daily average exposure, respectively. The hand-to-mouth route was the main way to reduce the average daily exposure risk of heavy metals in the study area. The total non-carcinogenic risk HI of the seven heavy metal elements ranked Mn > Co > Pb > Zn > Cr > Cu > Cd, and only Mn’s HI was greater than one. In this study, Zn, Cd, and Pb were comprehensively significant pollution factors, but they had a low health risk index that indicated a low significant health risk for adults. The non-carcinogenic and carcinogenic risk indices of all heavy metal elements, excluding Mn, were within acceptable limits. Because the study area experienced heavy winds and sand with scarce rainfall throughout the year, we recommend that areas with frequent industrial activities take protective measures such as controlling the speed of vehicles, especially large ones that transport production materials and heavy loads. Vehicles should be equipped with dust suppression devices to effectively prevent the spread of pollutants from the root causes.

This study used a different perspective and combined a variety of evaluation methods to explore the pollution characteristics and health risks of heavy metals in the dust fall of the desert steppe. Our results provide a scientific basis for the atmospheric, ecological, and environmental protection of this region.

## Supplemental Information

10.7717/peerj.12430/supp-1 Supplemental Information 1 Raw data. *Corresponding author e-mail: 705484905@qq.com (Nan Mi).

## Additional Information and Declarations

### Data Availability

The following information was supplied regarding data availability: The raw measurements are available as a Supplemental File.
